# Study on the Regulation Effect of Optogenetic Technology on LFP of the Basal Ganglia Nucleus in Rotenone-Treated Rats

**DOI:** 10.1155/2021/9938566

**Published:** 2021-07-28

**Authors:** Zongya Zhao, Yanxiang Niu, Peiqi Chen, Yu Zhu, Liangliang Shi, Xuewei Zhao, Chang Wang, Yehong Zhang, Zhixian Gao, Wenshuai Jiang, Wu Ren, Renjun Gu, Yi Yu

**Affiliations:** ^1^The Second Affiliated Hospital of Xinxiang Medical University, China; ^2^School of Medical Engineering of Xinxiang Medical University, China; ^3^Henan Province Neural Sensing and Control Engineering Technology Research Center, China; ^4^Henan Key Lab of Biological Psychiatry, Xinxiang Medical University, China

## Abstract

**Background:**

Parkinson's disease (PD) is a common neurological degenerative disease that cannot be completely cured, although drugs can improve or alleviate its symptoms. Optogenetic technology, which stimulates or inhibits neurons with excellent spatial and temporal resolution, provides a new idea and approach for the precise treatment of Parkinson's disease. However, the neural mechanism of photogenetic regulation remains unclear.

**Objective:**

In this paper, we want to study the nonlinear features of EEG signals in the striatum and globus pallidus through optogenetic stimulation of the substantia nigra compact part.

**Methods:**

Rotenone was injected stereotactically into the substantia nigra compact area and ventral tegmental area of SD rats to construct rotenone-treated rats. Then, for the optogenetic manipulation, we injected adeno-associated virus expressing channelrhodopsin to stimulate the globus pallidus and the striatum with a 1 mW blue light and collected LFP signals before, during, and after light stimulation. Finally, the collected LFP signals were analyzed by using nonlinear dynamic algorithms.

**Results:**

After observing the behavior and brain morphology, 16 models were finally determined to be successful. LFP results showed that approximate entropy and fractal dimension of rats in the control group were significantly greater than those in the experimental group after light treatment (*p* < 0.05). The LFP nonlinear features in the globus pallidus and striatum of rotenone-treated rats showed significant statistical differences before and after light stimulation (*p* < 0.05).

**Conclusion:**

Optogenetic technology can regulate the characteristic value of LFP signals in rotenone-treated rats to a certain extent. Approximate entropy and fractal dimension algorithm can be used as an effective index to study LFP changes in rotenone-treated rats.

## 1. Introduction

Parkinson's disease is a common neurological degenerative disease, and its main cause is the loss of the meso-black dopamine neurons in the middle brain, resulting in a decrease in the neurotransmitter of dopamine [1–4]. The clinical symptoms of Parkinson's disease include motor and nonmotor symptoms, such as slow movement, static tremors, abnormal body movement patterns, anxiety, depression, and sleep disorders [5–11]. At present, Parkinson's disease is mainly treated with drugs, but drug therapy is usually accompanied by serious side effects [12–15]. With the clinical application of deep brain stimulation (DBS), the treatment of Parkinson's patients has also led to new attempts to improve the survival rate of patients with Parkinson's disease compared to drug therapy [16]. Although DBS is becoming more and more effective in clinical application, the understanding of its treatment mechanism is still very limited, which limits the further clinical development of DBS [17–19].

Optogenetic technology transports light-sensing genes to specific brain areas and infects target neurons through a carrier. The photoreceptor protein is expressed on the cell membrane of the target neuron through reverse transcription. Compared with traditional electrical stimulation, optogenetic technology has high-quality characteristics such as space specificity, time specificity, and precise targeting [20]. Lee et al. characterized the different stages of Parkinson's disease by stimulating the striatum of rats and found that different parameters of illumination can make rats show different stages of Parkinson's symptoms [18]. In the experiment where photoacoustic stimulation of the brain increases the somatosensory pulse discharge response, moderate-intensity photoacoustic stimulation can increase the cortical somatosensory response to beard stretching [21]. Using optogenetic technology to inhibit GABA neurons in the globus pallidus can effectively improve dyskinesia of PD rats, which also indicates that the excessive activity of GABA neurons in the globus pallidus is the neural basis of involuntary movement [22]. In the experiment of controlling the light generation of the basal ganglia circuit to regulate Parkinson's motor behavior, Mikell and McKhann injected 6-OHDA bilaterally into the dorsal striatum with ChR2. One week later, dopaminergic neurons in this area are almost completely lost, accompanied by Parkinson's dyskinesias, including slow movement, reduced walking time, reduced motor initiation, and increased freezing. They stimulated the dorsal striatum with blue light and found that the direct pathway activation completely returned to the prelesion level, showing that the direct pathway activation eliminated motor retardation, increased motor activation, and reduced freezing [23].

At present, the research on PD rats' LFP with optogenetic technology mainly starts from the basal ganglia circuit. The globus pallidus and striatum are a common goal of neuromodulation in the treatment of Parkinson's disease and are also the final common output node of direct and indirect pathways in the basal ganglia [24]. A growing body of evidence shows that dyskinesias in patients with Parkinson's disease are associated with abnormally increased basal ganglia *β* frequency and decreased *γ* frequency oscillation activity [25–28]. Therefore, *β* oscillation is currently a potential biomarker for Parkinson's disease [29–31]. Beck et al. recorded LFP signals from the subthalamic nucleus and primary motor cortex of the reserpine model and 6-OHDA model. It was found that both models showed higher *β* frequency oscillations [32]. In order to understand how the lack of dopamine interferes with the electrical activity of the subcortical neural network, Wang et al. explored the effect of dopamine neuron depletion on the electrical activity of the primary motor cortex (M1) and the outer and inner parts of the pallidus (GPe and GPi). Comparing the local field potentials (LFPs) of rats with unilateral hemisphere dopamine neuron deletion with intact rats, it was found that the enhanced oscillatory activity and the enhanced synchronization of LFPs may cause movement disorders in the Parkinson rat model [33]. In order to determine the effect of dopamine depletion on the oscillatory activity of the thalamus-striatum pathway, they compared the control group and unilateral dopamine-injured rats and recorded LFPs from the parafascicular nucleus and dorsal side while rats were at rest or walking on the treadmill. It was found that the transition of the Parkinson rat model from the resting to moving state is related to different PF neuron types and increased LFPs and spike synchronization [34]. Although many discoveries have confirmed abnormal *β* and *γ* oscillations, the direct relationship between decreased dopamine levels in the basal ganglia and enhanced *β* and *γ* oscillations remains unclear [35].

In this study, the application of optogenetic technology in Parkinson's disease needs to be further studied. These previous studies on the treatment of Parkinson's disease by optogenetic technology are based on macroscopic behavioral studies, but the changes of brain LFP in Parkinson's disease rats before, during, and after laser stimulation are unknown. Therefore, we studied the effect of light stimulation on the LFP signals from the globus pallidus and striatum in the rotenone-treated rat model in the present study, which might provide theoretical support for clinical treatment and evaluation of Parkinson's disease.

## 2. Material and Method

### 2.1. Experimental Animals

In this study, 60 adult male SD rats weighing between 280 and 320 g provided by the Animal Center of Xinxiang Medical University were selected. SD rats are kept in an animal room with alternating light and dark for 12 hours with suitable temperature and humidity. The rats can freely take water and food. The whole group is divided into two groups, the control group and the experimental group. The experimental group is injected with the virus after successful modeling. One part of the brain slice is taken to observe the virus expression, and the other part is implanted with a photoelectrode for light therapy. According to the position of the implanted electrode, it is divided into the pallidus group and striatum group. During the entire experiment, all operations were approved by the Animal Ethics Society of Xinxiang Medical University and performed in accordance with the international experimental animal use ethics standards. During the entire experiment, the number of animals used and the suffering of the animals were reduced as much as possible.

### 2.2. Main Instruments and Reagents

The main instruments are the stereoscopic locator purchased from RWD (Life Science Co., Shenzhen, China) and Cerebus acquisition system purchased from Black Rock Company (USA). The reagents are rotenone purchased from Sigma Corporation (St. Louis, Missouri, USA), apomorphine purchased from Sigma Corporation (St. Louis, Missouri, USA), metformin purchased from Sigma Corporation (St. Louis, Missouri, USA), viral architecture pAAV-CaMKIIa-hChR2(H134R)-mCherry purchased from Obio Technology, Shanghai, China, 0.3% sodium pentobarbital, PBS solution, penicillin, 18% sucrose aqueous solution, and 4% polyformaldehyde solution; all reagents are configured on the day of the experiment.

### 2.3. Preparation of the Rat Model of Parkinson's Disease

The experimental group was injected with rotenone into the substantia nigra compact (SNC) and midbrain ventral tegmental area (VTA) on the right side of SD rats to establish a unilaterally completely damaged rotenone-treated model. The control group was injected with the same dose of dimethyl sulfoxide (DMSO) at the same location. Rats were anesthetized by intraperitoneal injection of sodium pentobarbital (0.3%) at 1 ml/100 g. According to rat brain atlas [36], the coordinates of substantia nigra compact area are as follows: anteroposterior (AP): -5 mm, mediolateral (ML): 2 mm, and dorsoventral (DV): 7.9 mm, and the coordinates of ventral tegmental area are as follows: AP: -4.92 mm, ML: 1 mm, DV: 8.4 mm. Using a stereotaxic device, 2 *μ* l rotenone was injected into each target brain area through a 25 *μ*l microsyringe. During injection, the needle should be inserted at 1 mm/min. After reaching the target brain area, the needle should be retained for 5 minutes and the needle should be withdrawn slowly at 1 mm/min to prevent drug backflow [37]. After making the model, intraperitoneal injection of penicillin was given to the rat in an amount of 0.5 ml daily to prevent infection. Three weeks after the rats were modeled, the rats in the experimental group and the control group were induced with apomorphine every night and the prepared apomorphine solution was injected intraperitoneally at the amount of 1 ml/kg. The rotenone-treated rats were believed to be successful if they showed symptoms such as stiff tail, arched back, frequent head nodding, erect hair, tremor, unsteady walking, and fixed rotation to the contralateral side more than 7 times per minute 5–30 min after injection.

### 2.4. Virus Injection

The virus was injected into the rats with PD. The selected viral framework was pAAV-CaMKIIa-hChR2(H134R)-mCherry. The virus carried the red fluorescent gene (mCherry) and the light-sensing gene hChR2 (H134R). After the virus was expressed in the host cell, the mCherry gene will express a fluorescent protein on the host cell, which was red when observed under a fluorescent microscope, so we can also judge whether the virus was successfully expressed based on it. The light-sensing gene hChR2 (H134R) was a light-sensitive ion channel protein gene that can be activated by blue light. It expressed a hChR2 protein on the cell membrane of dormitory cells. The channel protein was opened under 470 nm blue light. At this time, the host cell membrane changes in potential and the cell was activated after reaching the action potential, which was also a core point of this experiment. The final titer of the virus was 2.01 × 10^13^ *μ*g/ml. In brief, the purification table was sterilized under ultraviolet for 30 minutes, the virus melted on the ice, and then, 1 *μ*l of the melted virus was collected in a microsyringe, and 1 *μ*l/min was injected into the SNC (AP: −5 mm, ML: 2 mm, and DV: 7.9 mm). This protocol was the same as the one used to establish the rat PD model. The whole protocol was performed in the purification table.

### 2.5. Brain Tissue Collection and Processing

Two rats were randomly selected after modeling and after injecting virus. Firstly, the brains were perfused with 250 ml paraformaldehyde (4%) solution. Secondly, the taken-out brain was fixed in paraformaldehyde (4%) solution and dehydrated with 15% and 30% sucrose solution. Thirdly, the substantia nigra dense part was sliced into coronal slices at a thickness of 30 *μ*m in a cryostat (Leica Microsystems, Spain) and stored in the refrigerator at −20 degrees. Fourthly, take the brain tissue section out and rewarm at room temperature for 10 minutes and permeate with 0.3% triton solution for 15 minutes. Fifthly, put the permeable film into citric acid solution for 15 minutes at 98 degrees, block with serum goat serum for 20 minutes, and add no. 1 antibody (T1299 (1 : 10000 dilution)) 30 *μ*l–50 *μ*l to the tissue. Sixthly, put the slices in the refrigerator at 4 degrees for 12–18 h. The last steps were adding no. 2 antibody (488 (1 : 500 dilution)) 30 *μ*l–50 *μ*l to the tissue, putting the section in a refrigerator at 4 degrees overnight, and adding 50 *μ*l fluorescent dye DAPI to the tissue for staining for 5 minutes and sealing it.

### 2.6. Fabrication of Microwire Array Electrode

**Figure 1:**
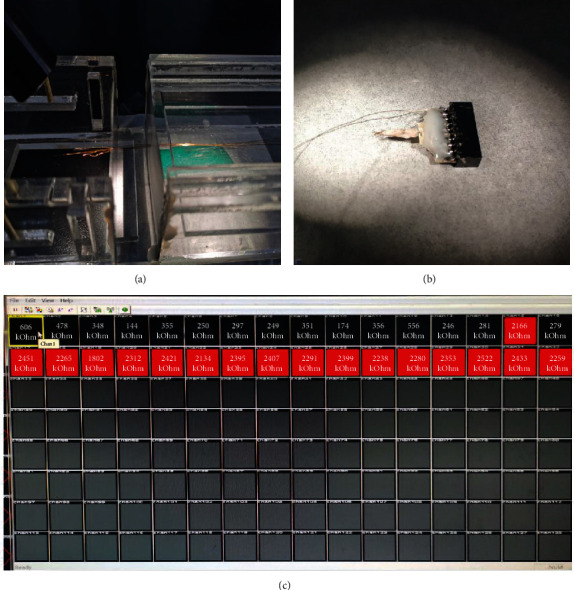
(a) Electrode mold. The electrode wire (12–30 *μ*m in diameter) needs to be manually passed through the mold to form a 4∗8 array, and the distance between the two electrode wires is 200–300 *μ*m. (b) A completed electrode. (c) Electrode impedance measurement; red represents a large impedance.

Using a stereo microscope, thread 16 nickel-chromium alloy electrode wires into the mold made of the polycarbonate (PC) (Figure 1(a)). The electrode wires are arranged in 4 rows and 4 columns, and each electrode wire has a diameter of 130 *μ*m. After wearing them, the circuit board is also fixed to the mold and then encapsulated with polyethylene glycol. After the polyethylene glycol is solidified, the electrode wires were welded with the contact points on the circuit board. Finally, two silver wires were welded on the circuit board as the reference and ground electrode. The fabricated electrode (Figure 1(b)) is used to test its own impedance with the Cerebus signal acquisition system. The software under the Cerebus system can detect the impedance value under 1 kHz (the firing frequency of neurons); the results are shown in Figure 1(c).

### 2.7. Acquisition of Rat LFP Signals

According to the different locations of implanted electrodes, the remaining rats after the morphological experiment were divided into two groups for experiment. The specific operation steps are as follows:

Firstly, half of the rat model is anesthetized with 0.3% sodium pentobarbital by 1 ml/100 g intraperitoneal injection. After the rats were anesthetized, they are fixed on the stereotaxic apparatus and the sutured wounds are cut. Four small holes were grinded out on the skull for placing screws, which are used to fix the electrodes. According to the brain atlas, the position of the globus pallidus is AP: −0.96 mm, ML: 3 mm, and DV: −5.9 mm. Then, the small holes were clean under a microscope, the dura mater and pia mater were open, and the 16-channel microwire electrodes were slowly implanted into the globus pallidus brain area. During this period, the Cerebus acquisition system was used to observe the signal at the same time. After that, the optical fiber was implanted into the substantia nigra dense part and the optical fiber was fixed with the electrode through dental tray cement. Then, the rats were kept in the animal room with injection of penicillin (800000 units) once a day for 5 consecutive days. Rats in the control group were treated with electrodes in the same part.

Secondly, half of the rat model is anesthetized with 0.3% sodium pentobarbital by 1 ml/100 g intraperitoneal injection. After the rats were anesthetized, they are fixed on the stereotaxic apparatus and the sutured wounds are cut. Four small holes were grinded out on the skull for placing screws, which are used to fix the electrodes. According to the brain atlas, the position of the striatum is AP: 0.8 mm, ML: −3.2 mm, and DV: −5.2 mm. Then, the small holes were clean under a microscope, the dura mater and pia mater were open, and the 16-channel microwire electrodes were slowly implanted into the striatum brain area. During this period, the Cerebus acquisition system was used to observe the signal at the same time. After that, the optical fiber was implanted into the substantia nigra dense part and the optical fiber was fixed with the electrode through dental tray cement. Then, the rats were kept in the animal room with injection of penicillin (800000 units) once a day for 5 consecutive days. Rats in the control group were treated with electrodes in the same part.

### 2.8. Optogenetic Experiment

**Figure 2:**
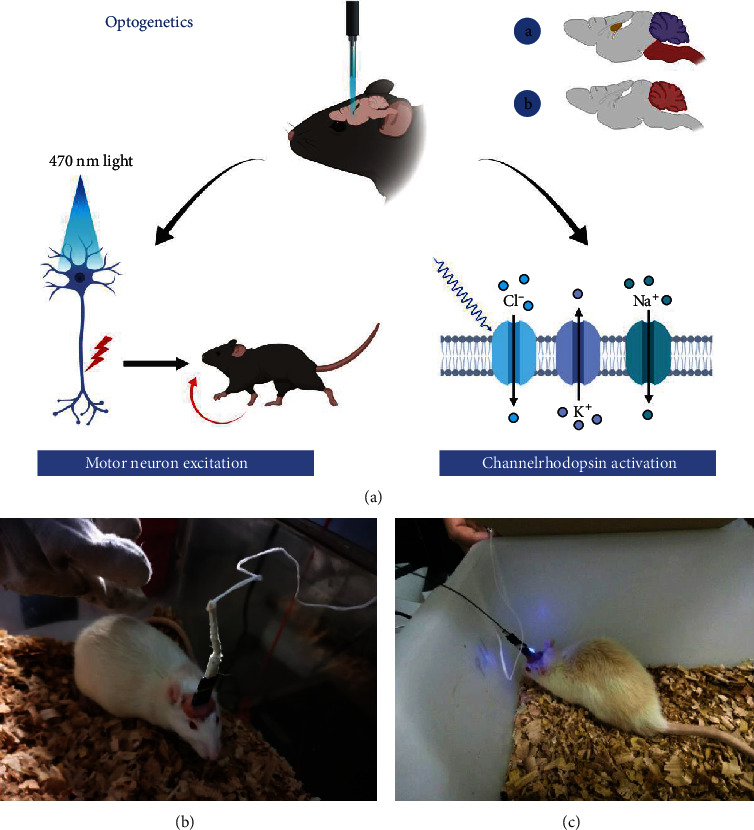
(a) The whole process of optogenetic expression. After virus injection, the channel protein was opened under 470 nm blue light irradiation. At this time, the ions in the host cell flow across the membrane, causing the neurons to excite. Photograms of the rat with laser (b) off and (c) on.

One week later, an optogenetic experiment (Figure 2(a)) was performed and the rat LFP was collected with the Cerebus acquisition system at a sampling frequency of 1000 Hz. Before the experiment, the experimental rats were placed in the laboratory for 3 days so that the experimental rats could get used to the laboratory environment. Our experimental plan is as follows: we adjust the power of the blue laser generator to 1 mW and the frequency to 25 Hz and start the experiment at 7 o'clock every night. The LFP signals were recorded from each rat before, during, and after light stimulation 1 minute each time and collected 10 times continuously (Figures 2(b) and 2(c)). Ensure that the rats were in a quiet state for at least 1 min in total as valid data.

### 2.9. LFP Analysis

**Figure 3:**
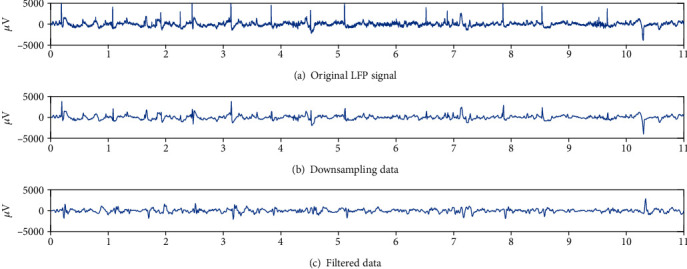
Randomly selected one channel, taking 11 seconds of LFP data to analyze and exhibit.

**Figure 4:**
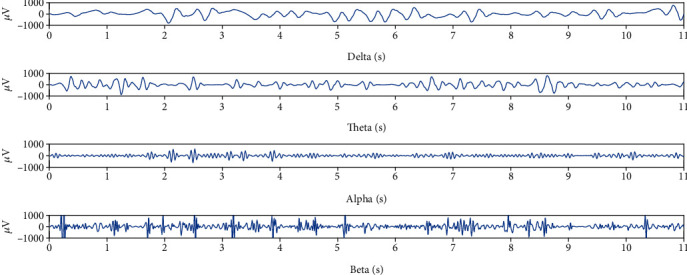
After removing the artifacts such as EMG and EOG, the useful signals were obtained. After wavelet packet function decomposition and reconstruction, the LFP signals of four bands (*δ*, *θ*, *α*, and *β*) were obtained for the next step of feature calculation.

In this study, we analyzed the local field potential. Firstly, the original signal (Figure 3(a)) was downsampled at a frequency of 128 Hz (Figure 3(b)), and then, a bandpass filter of 3–35 Hz was used to extract the useful frequency band (Figure 3(c)), and finally, some artifacts such as EMG and oculus were removed to obtain useful signals. After the preprocessing, the LFPs were decomposed by using wavelet packet function and then reconstructed in [4 0], [4 1], [4 2], [4 3] + [3 2] + [3 3] to a get delta (0.5–3.5 Hz) wave, theta (4–7 Hz) wave, alpha (8–13 Hz) wave, and beta (14–30 Hz) wave (Figure 4). The LFP signal is a nonstationary random signal, so we used the classical nonlinear dynamic analysis algorithm such as the fractal dimension and approximate entropy fast algorithm to analyze the collected LFP signal. The steps of the approximate entropy fast algorithm were performed following the formula described as follows:

Given an *N* point time series, the *N*∗*N*, a two-value matrix *D* was calculated. Elements in *D* are *d*_*ij*_:

(1) $$\begin{array}{c} d_{ij}=\left\{\begin{array}{c} 1\;\left|u\left(i\right)-u\left(j\right)\right|<r,\\ \begin{array}{cc} 0 & \left|u\left(i\right)-u\left(j\right)\right| \end{array}\geq r, \end{array}\right.\;1\leq i\leq N,1\leq j\leq N. \end{array}$$

Calculate *C*_*i*_^2^(*r*) and *C*_*i*_^3^(*r*).

(2) $$\begin{array}{c} c_{i}^{2}\left(r\right)=\overset{N-1}{\underset{j=1}{\sum }}d_{ij}\cap d_{\left(i+1\right)\left(j+1\right)},\\ C_{i}^{3}\left(r\right)=\overset{N-2}{\underset{j=1}{\sum }}d_{ij}\cap d_{\left(i+1\right)\left(j+1\right)}\cap d_{\left(i+2\right)\left(j+2\right)}. \end{array}$$

According to the fourth step of the approximate entropy algorithm, *ϕ*^2^(*r*) and *ϕ*^3^(*r*) are obtained.

(3) $$\begin{array}{c} \phi^{m}\left(r\right)=\frac{1}{N-m+1}\overset{N-m+1}{\underset{i=1}{\sum }}\ln C_{i}^{m}\left(r\right). \end{array}$$

Finally, approximate entropy is calculated.

(4) $$\begin{array}{c} \text{ApEn}\left(2,r\right)=\phi^{2}\left(r\right)-\phi^{3}\left(r\right). \end{array}$$

The algorithm steps of fractal dimension were performed following the formula described as follows:

*ε* is the edge of the box, and *N*(*ε*) is the number of fractals covered by the box. This algorithm determines the fractal dimension of the fractal body by covering the fractal body with a small box whose edge length is *ε*.

(5) $$\begin{array}{c} D=\underset{\varepsilon ⟶0}{\lim}\left[\left|\frac{\log_{2}\left(N\left(\varepsilon \right)\right)}{\log_{2}\left(1/\varepsilon \right)}\right|\right]. \end{array}$$

*N*(*ε*) is the total number of small boxes covering the fractal body under test. *x*_*ε*_ is a time series with length *L*, and fractal dimension *D* is the ratio of log_2_(*N*(*ε*)) to log_2_(1/*ε*).

(6) $$\begin{array}{c} \ln_{\varepsilon }\left(i\right)=\frac{\left(\max\left(x_{\varepsilon }\right)-\min\left(x_{\varepsilon }\right)\right)}{\varepsilon_{i}},\\ \left.\varepsilon \in 2^{k}\right|k=1,2,\cdots ,\log_{2}\left(L\right)-1,\\ N\left(\varepsilon \right)=\underset{i}{\sum }n_{\varepsilon }\left(i\right). \end{array}$$

### 2.10. Statistical Analysis

SPSS19.0 software was used for data analysis; measurement data was expressed as mean ± standard deviation. First, perform the Kolmogorov-Smirnov (KS) test on the data and the result accords with the normal distribution. Then, after analyzing various factors, 12 sets of data (4 control groups, 4 striatum groups, and 4 globus pallidus groups) were finally obtained for statistical analysis. The independent sample *t*-test was used for comparison between groups, and repeated measurement analysis of variance was used for comparison within groups. *p* < 0.05 for all data was considered statistically significant.

## 3. Results

### 3.1. Parkinson Rat Model

**Figure 5:**
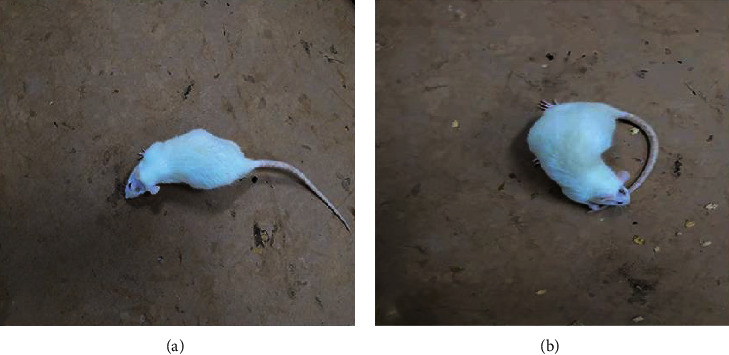
Parkinson's disease model validation. Apomorphine was intraperitoneally injected, and the rat was placed in a square box. After 5–30 minutes, the rat can be observed to rotate to the contralateral side of the modeled side > 7 laps/min: (a) the unsuccessful rat model; (b) the successful model building.

The rotenone-treated rats were injected intraperitoneally with apomorphine at 1 ml/kg to induce the symptoms of Parkinson's disease. We found that 16 of the 40 rats had stiff tails, arched backs, frequent tails, nodding, erect hair, tremor, unsteady walking, fixed rotation to contralateral side more than 7 times per minute, and other symptoms [38] (Figures 5(a) and 5(b)), and these 16 rotenone-treated rat models were finally determined to be successful.

### 3.2. Morphological Analysis

**Figure 6:**
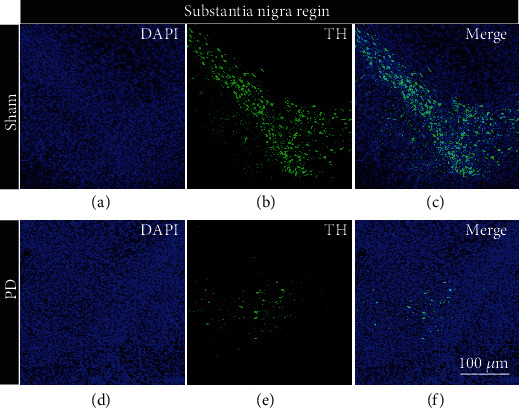
Modeling success. (a–c) The pictures are the coronal sections of the substantia nigra compact part (AP: −5 mm, ML: 2 mm, and DV: 7.9 mm) of the control group rat under different conditions (DAPI, TH, and merge). (d–f) The pictures are the coronal sections of the SNC part (AP: −5 mm, ML: 2 mm, and DV: 7.9 mm) of the experimental group rat under different conditions (DAPI, TH, and merge). Scale bar 100 *μ*m.

Two successfully modeled rats and two rats injected with the virus were randomly selected, and their brain tissues were taken out for frozen section and immunofluorescence staining. The rat brain area analyzed by immunohistochemistry was the substantia nigra dense part. TH is a specific staining index of dopaminergic neurons. The higher the positive rate of immunohistochemical staining, the greater the number of dopaminergic neurons. By observing the immunohistochemical staining of the control group in Figures 6(a)–6(c) and the experimental group in Figures 6(d)–6(f), the number of neurons in the dense brain area of the rat substantia nigra was lost in large numbers, morphologically verifying the success of the model.

**Figure 7:**
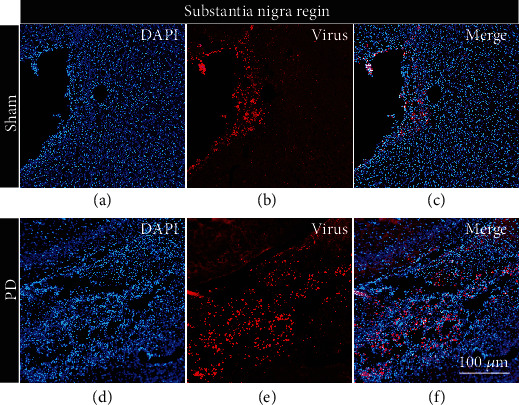
Viral expressed. (a–c) The pictures are the coronal sections of the substantia nigra compact part (AP: −5 mm, ML: 2 mm, and DV: 7.9 mm) of the control group rat under different conditions (DAPI, TH, and merge). (d–f) The pictures are the coronal sections of the SNC part (AP: −5 mm, ML: 2 mm, and DV: 7.9 mm) of the experimental group rat under different conditions (DAPI, TH, and merge). Scale bar 100 *μ*m.

Because the virus comes with a red marker gene, it will express a red fluorescent protein on the remaining dopaminergic neurons after normal expression. Our results showed that red fluorescence appeared in the dopaminergic neurons in the substantia nigra compact area on the right (Figure 7), while the absence of red fluorescence in other brain areas indicated that the virus was successfully expressed on dopaminergic neurons.

### 3.3. Analysis of LFP Signals in the Rat Striatum

**Figure 8:**
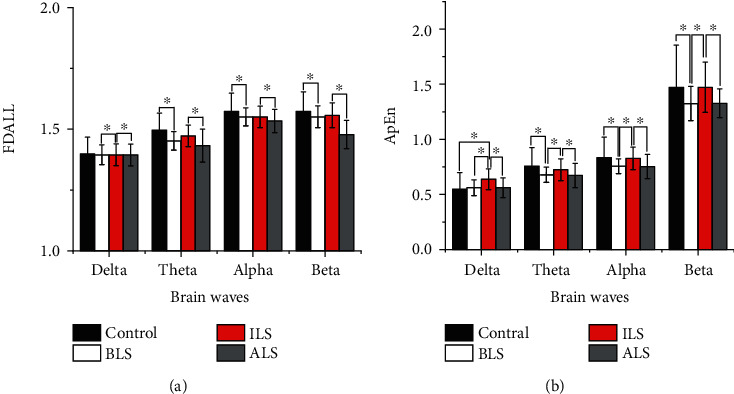
The characteristic value of the LFP signal in the rat striatum brain area after optogenetic experiment. (a) Fractal dimension and (b) approximate entropy. Control represents the LFP signal of rats in the control group; BLS represents the LFP signal before light stimulation in rotenone-treated rats; ILS represents the LFP signal during light stimulation in rotenone-treated rats; ALS represents light in rotenone-treated rat LFP signal after stimulation.

Approximate entropy and fractal dimension algorithm were used to calculate the characteristic values of the LFP signals of the control group and experimental group rats before, during, and after the light stimulation. The results are shown in (Figure 8).

Results showed that the approximate entropy and fractal dimension of the control group are all larger than those of PD rats before light stimulation in all four frequency bands and the approximate entropy of control group and rotenone-treated rats before light stimulation showed statistical differences (*t* = 6.313, *p* < 0.001) in all four frequency bands. However, the fractal dimension of the control group showed statistical difference with rotenone-treated rats before light stimulation only in the delta band (*t* = 5.215, *p* < 0.001). In addition, although the approximate entropy of the control group was greater than that of rotenone-treated rats during light stimulation for all frequency bands, there was no statistical difference (*t* = 0.373, *p* = 0.710) and the fractal dimension algorithm of four frequency bands during light stimulation in the control group also did not show statistical difference with Parkinson's disease rats (*t* = 1.80, *p* = 0.073). Repeated measures ANOVA showed that there existed statistical differences in characteristic values of the LFP signals before, during, and after light stimulation for Parkinson's disease rats (*F*(2,440) = 5.930, *p* = 0.03), and the LFP signal characteristics of four frequency bands during light stimulation were significantly greater than those before (*F*(1,191) = 10.229, *p* < 0.05) and after (*F*(1,220) = 8.229, *p* < 0.05) light stimulation. However, there is almost no difference in the characteristic values of the LFP signal in four frequency bands before and after light stimulation in Parkinson's disease rats (*F*(1,219) = 5.546, *p* = 0.052).

### 3.4. Analysis of the LFP Signal of the Rat Globus Pallidum

**Figure 9:**
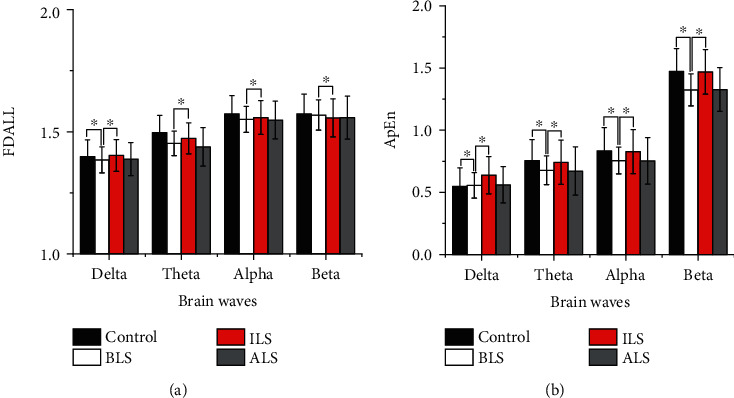
The characteristic value of the LFP signal in the rat globus pallidus brain area after optogenetic experiment. (a) Fractal dimension and (b) approximate entropy. Control represents the LFP signal of rats in the control group; BLS represents the LFP signal before light stimulation in rotenone-treated rats; ILS represents the LFP signal during light stimulation in rotenone-treated rats; ALS represents light in rotenone-treated rat LFP signal after stimulation.

Approximate entropy and fractal dimension algorithm were used to calculate the characteristic values of the LFP signals of the control group and experimental group rats before, during, and after the light stimulation. The results are shown in Figure 9.

Results showed that the fractal dimensions of the control group are all larger than those of PD rats before, during, and after light stimulation in all four frequency bands. However, only the fractal dimension feature values of the LFP signal in theta and alpha bands are statistically significant (*t* = 6.508, *p* < 0.001). The fractal dimensions of four frequency bands during light stimulation are all larger than those of PD rats before and after stimulation. However, the fractal dimension of PD rats during stimulation showed statistical difference with rotenone-treated rats before light stimulation only in the delta band (*F*(1.100,210.186) = 9.839, *p* < 0.001). Lastly, the fractal dimension of four frequency bands of PD rats during light stimulation showed statistical difference with rotenone-treated rats after light stimulation (*F*(1.243,237.496) = 10.386, *p* < 0.001).

It can be seen in Figure 8(b) that the approximate entropy feature values of the LFP signal in the theta, alpha, and beta bands of the control group are larger than those of the PD rats before stimulation and have statistical significance (*t* = 6.243, *p* < 0.001); however, the approximate entropy feature value of the LFP signal of the control group in the delta band is lower than that of the PD rats before stimulation and is not statistically significant (*t* = 1.518, *p* = 0.130). The approximate entropy feature values of the LFP signal in the theta, alpha, and beta bands of the control group were higher than the approximate entropy feature values of the LFP signal in the light stimulation of Parkinson's disease rats, which were not statistically significant (*t* = 0.940, *p* = 0.348). In addition, the approximate entropy of the control group in the delta was lower than that of PD rats during stimulation and has statistical significance (*t* = 2.198, *p* < 0.05). The approximate entropy values of four frequency bands during light stimulation are all larger than those of PD rats before and after stimulation and have statistical significance (*F*(2,382) = 7.165, *p* < 0.05). The approximate entropy characteristic values of LFP signals in the theta and alpha bands of rotenone-treated rats before light stimulation are higher than those of rotenone-treated rats after light stimulation. The approximate entropy characteristic values of LFP signals in the delta and beta bands before light stimulation in rotenone-treated rats were lower than those after light stimulation in rotenone-treated rats, and they were not statistically significant (*F*(1.953,373.092) = 9.839, *p* = 0.403).

## 4. Discussion

Parkinson's disease is a common degenerative disease of the nervous system. The main cause is the progressive loss of dopaminergic neurons in the substantia nigra of the midbrain, leading to a decrease in dopamine neurotransmitters. At present, the methods of building Parkinson's animal models include chemical drug models, biotoxic substance models, mechanical damage models, and genetic models. In this study, we found that the use of unilateral two-point injection of rotenone in SNC and VTA increased the success rate. Compared with the celiac injection or subneck injection of rattan ketone to make the Parkinson's disease rat model, stereoscopic positioning injection mold directly damages the target brain region and could shorten the molding time [39–42].

Parkinson's disease is mainly related to the basal ganglia circuit, so lots of researches focused on this circuit. Functionally, the basal ganglia circuit constitutes the “cortex-basal ganglia-thalamus” loop [43, 44], and there are three pathways related to Parkinson's disease in this loop including the direct pathway (cerebral cortex-striatum-internal globus pallidus-thalamus-cortex), indirect pathway (cerebral cortex-striatum-outer globus pallidus-subthalamic nucleus-inner globus pallidus-thalamus-cortex), and super direct pathway (nigrostriatal pathway). The direct pathway mainly plays a role of excitation regulation, and the indirect pathway plays a role of inhibition. In the basal ganglia loop, the globus pallidus and the striatum are important relay nuclei and these brain regions are also used in experiments to verify the treatment of Parkinson's disease by optogenetic technology. After injecting rotenone into SD rats, it was found that the number of neurons in the substantia nigra compact area was significantly reduced, resulting in a reduction in the number of dopamine released, and the total amount of neuronal discharge might also be reduced. Our research method is to stimulate the remaining dopamine neurons to treat Parkinson's disease.

In the present study, in addition to observing the Parkinson's model in behavioral assessment and immunohistochemistry, we focused on the LFP signal. In the indirect pathway of normal rats, the dopamine neurotransmitter secreted by the dopaminergic neurons in the substantia nigra compact was released into the striatum, which inhibited the related D2-type *γ*-aminobutyric acid neurons in the pathway. After the occurrence of Parkinson's disease, the number of dopaminergic neurons in the substantia nigra compact area decreases, resulting in a decrease in the dopamine neurotransmitter released into the striatum. The decrease in the dopamine neurotransmitter affects D2-type gamma-aminobutyric acid neurons. The inhibitory effect is weakened, resulting in abnormal excitement of D2-type gamma-aminobutyric acid neurons, and the excessive D2-type gamma-aminobutyric acid neurotransmitter is released to the outer globus pallidus, which inhibits the discharge activity of the outer globus pallidus, and these support our result that the characteristic values of LFP signals of normal rats are higher than those of rotenone-treated rats. When the laser is turned on, the activated light-sensitive protein channel expressed on the remaining dopaminergic neurons is opened and the dopaminergic neurons release nearly normal amounts of the dopamine neurotransmitter to the striatum, thereby returning the discharge activity of the lateral globus pallidus to the normal level.

In the direct pathway, the dopaminergic neurons in the dense brain area of the substantia nigra of patients are greatly reduced and the content of the dopaminergic neurotransmitter in the striatum brain area is lower than normal. The decrease in the dopaminergic neurotransmitter weakens the activation of D1-type *γ*-aminobutyric acid neurons in the direct pathway, resulting in a decrease in the excitement of D1-type *γ*-aminobutyric acid neurons, which deinhibits the substantia nigra reticulum/medial globus pallidus, suppresses the thalamus, and reduces the secretion of glutamate. The reduction of the dopamine neurotransmitter weakens the inhibitory effect of D2-type gamma-aminobutyric acid neurons in the indirect pathway, resulting in increased inhibition of gamma-aminobutyric acid and enkephalin, which inhibits the lateral globus pallidus, excites subthalamic nucleus and substantia nigra reticulum/medial globus pallidus, and finally inhibits the thalamus. Glutamate is an excitatory neurotransmitter, and reduction of glutamate in the striatum from the subthalamic nucleus and the motor cortex leads to reduced activation of striatal neurons [45, 46].

Therefore, the characteristic values of the LFP signals in the delta, theta, alpha, and beta bands in the control group are greater than the LFP signal characteristic values of the rotenone-treated rats before light stimulation. When the laser stimulates the dopaminergic neurons in the dense part of the substantia nigra, the dopaminergic neurons release a large amount of dopamine neurotransmitter, making the dopamine content in the striatum reach or close to the normal level. Therefore, neurons in the striatum discharge normally or are close to normal. The characteristic values of the LFP signals in the delta, theta, alpha, and beta bands before light stimulation in rotenone-treated rats are smaller than those during light stimulation of rotenone-treated rats. The characteristic values of the LFP signals in the delta, theta, alpha, and beta bands during the light stimulation of rotenone-treated rats are close to those of normal rats. The change of the LFP characteristic value verifies the existence of the direct pathway and indirect pathway to a certain extent.

In Figures 8 and 9, it can be seen that the characteristic values of LFP signals in rotenone-treated rats during light stimulation are greater than those in rotenone-treated rats after light stimulation. The characteristic values of the LFP signal in the four frequency bands after light stimulation are smaller than those in rotenone-treated rats before stimulation. Since the interval between during light and after is 2 hours, the dopamine neurotransmitter released into the striatum during the light stimulation is gradually consumed and returned to the level before the light stimulation. Therefore, the characteristic value of the LFP signal during light stimulation is statistically different from that of rotenone-treated rats after light. There is no statistical difference between the characteristic value after light stimulation of rotenone-treated rats and that of rotenone-treated rats before light stimulation. It can be seen in Figures 8 and 9 that there is no statistical difference in the delta band LFP signal characteristic values between control rats and rotenone-treated rats. The main pathological change of Parkinson's disease is the damage of the substantia nigra. In addition, locus coeruleus, dorsal vagus nucleus, innominate substance, sympathetic nerves, and limbic lobes may also be damaged [47, 48]. Nagano-Saito et al. found that the atrophy of the cerebral cortex in PD patients is mainly seen in the limbic system, paralimbic system, and prefrontal cortex through imaging examinations [49]. Kenny et al. found that the frontal lobe of PD patients was significantly atrophied, including the right superior frontal gyrus, middle frontal gyrus, and inferior frontal gyrus [50]. Deng et al. found that PD patients with mild cognitive impairment showed a significant decrease in anisotropy in white matter of the left frontal lobe, the right temporal lobe, and the bilateral anterior cingulate bundles [51]. Patients with Parkinson's disease are often accompanied by cognitive dysfunction, and studies have found that PD patients' cognitive impairment is related to the frontotemporal cortex, subcortical atrophy, and delta wave abnormalities [52, 53], which is consistent with the findings of this study.

However, this experiment also has certain limitations. In the experiment, a single parameter was used for the light stimulation parameter and no attempt was made to find the best parameter for the treatment of Parkinson's disease by optogenetic technology. In future research, several more parameters can be set to try the therapeutic effect of optogenetic technology on Parkinson's disease under different parameters.

## 5. Conclusion

In this study, optogenetic technology was used to control the secretion of the dopamine neurotransmitter in the substantia nigra compact area and to observe the changes in brain electrical signals in the striatum and globus pallidus. The results show that optogenetic technology has an effect on the LFP signals of the basal ganglia nucleus of PD rats.

## Data Availability

The datasets used and analyzed in the current study are available from the corresponding author upon reasonable request.
